# Interhemispheric effects of iTBS on the fronto-parietal network: Evidence from dual-site stimulation

**DOI:** 10.1016/j.ynstr.2025.100744

**Published:** 2025-07-10

**Authors:** Isabell Int-Veen, Beatrix Barth, Ramona Täglich, Betti Schopp, Hans-Christoph Nuerk, Christian Plewnia, Stefanie De Smet, Marie-Anne Vanderhasselt, Andreas J. Fallgatter, Ann-Christine Ehlis, David Rosenbaum

**Affiliations:** a Department of Psychiatry and Psychotherapy, Tübingen Center for Mental Health (TüCMH), University of Tübingen, Tübingen, Germany; b German Center for Mental Health, Partner site Tübingen, Germany; c LEAD Graduate School & Research Network, University of Tübingen, Tübingen, Germany; d Department of Psychology, University of Tübingen, Germany; e Department of Head and Skin, Psychiatry and Medical Psychology, Ghent University Hospital, Ghent University, Ghent, Belgium; f Ghent Experimental Psychiatry (GHEP) lab, Ghent, Belgium; g Brain Stimulation and Cognition (BSC) Lab, Department of Cognitive Neuroscience, Faculty of Psychology & Neuroscience, Maastricht University, Maastricht, the Netherlands

**Keywords:** Theta Burst Stimulation, Stress, Trier Social Stress Test, VLPFC, DLPFC

## Abstract

This study investigated the neuromodulatory effects of Theta Burst Stimulation (TBS) on resting-state functional connectivity (FC) following psychosocial stress induced by the Trier Social Stress Test (TSST). Given its key role in cognitive control and emotion regulation — processes highly relevant for rumination — we focused on the frontoparietal network. Across two studies, intermittent (iTBS) and continuous (cTBS) protocols were applied to the left Dorsolateral Prefrontal Cortex (DLPFC; study 1) and right Ventrolateral Prefrontal Cortex (VLPFC; study 2) prior to stress induction. Functional Near-Infrared Spectroscopy (fNIRS) was used to assess neural changes. A total of 88 (study 1) and 89 (study 2) healthy participants were recruited, balanced for low and high trait rumination. Each participant received both active and sham TBS (iTBS or cTBS), in a randomized, counterbalanced design. Results indicated that iTBS elicited excitatory effects on prefrontal and fronto-parietal connectivity, whereas cTBS effects were more variable. Trait rumination emerged as a modulator of TBS effects: In study 1, significant interactions for FC between the right VLPFC and Somatosensory Association Cortex (SAC) when stimulating the left DLPFC emerged, while study 2 revealed similar interactions for FC between the left DLPFC and SAC and intra-SAC FC when stimulating the right VLPFC. Correlations between post-stress state rumination and FC changes further support these findings. These results underscore the importance of neural assessments in TBS research and highlight the complexity of individual differences in state and trait rumination. Understanding the interplay between TBS, fronto-parietal connectivity, and rumination may provide valuable insights into personalized neuromodulation strategies.

## 1 Introduction

“Repetitive, prolonged, and recurrent thought about one's self, one's concerns and one's experiences is a mental process commonly engaged in by all people” (Watkins, 2008, p. 163). This phenomenon, referred to as repetitive negative thinking (RNT), includes ruminative thoughts and worry, among others. While RNT is a common experience in healthy individuals, it has been identified as a risk factor for the development of mood disorders such as depression and anxiety, as well as other mental health conditions. For this reason, it is considered a transdiagnostic cognitive vulnerability (Nolen-Hoeksema et al., 2008). Prolonged negative focus on oneself, concerns and experiences is associated with enhanced negative affect, psychopathological symptoms and actively interferes with effective problem-solving and instrumental behavior, constituting a vicious cycle of negative affect and negative cognition, increasing the likelihood that initial symptoms of a corresponding (subclinical) mental disorder will worsen and, for instance, develop into episodes of major depression (for comprehensive reviews, see e.g. Nolen-Hoeksema et al., 2008; Watkins and Roberts, 2020).

A recent attempt to summarize the perpetuating factors underlying mechanisms of rumination proposes the H-EX-A-GO-N (Habit development, EXecutive control, Abstract processing, GOal discrepancies, Negative bias) model by Watkins and Roberts (2020). What is particularly interesting in this model is that neurobiological findings, namely patterns of brain activation that have been associated with rumination, are easily integrated. Neurobiological research suggests that the neural basis of ruminative thinking is characterized by heightened activation and increased functional connectivity (FC) within regions of the Default Mode Network (DMN), which includes the medial prefrontal cortex, posterior cingulate cortex, and precuneus, among others (Hamilton et al., 2011; Jones et al., 2017; Mandell et al., 2014; Ray et al., 2005; Zhou et al., 2020). Generally, the DMN is implicated in abstract, self-referential processing and exhibits increased activation during resting-state measurements, while it is deactivated during goal-directed cognitive tasks.

Consistent with the H-EX-A-GO-N model (Watkins and Roberts, 2020), which posits that rumination is associated with deficits in executive control, alterations in the functioning of brain regions within the Fronto-Parietal Network (FPN) have been repeatedly observed. Notably, the FPN typically exhibits increased activation during goal-directed cognitive tasks, and regions associated with the FPN, such as prefrontal areas like the DLPFC, have also been implicated in ruminative thinking across different tasks and samples (Cooney et al., 2010; Kross et al., 2009; Rosenbaum et al., 2018a; Rosenbaum et al., 2018b; Vanderhasselt et al., 2011). It is important to note that all of the aforementioned neural changes are also largely associated with general depressive symptomatology (Menon, 2011; Mulders et al., 2015), making it difficult to disentangle the specific contributions of trait rumination and depressive symptoms to the neural underpinnings, as these are intuitively highly correlated, if not representing the same general negative cognitive-affective process in psychopathology.

A recent study aimed at distinguishing the neural correlates of rumination and depressive symptoms found that stress-induced hypoactivation in the left DLPFC and VLPFC was predicted by state rumination, rather than trait rumination or depressive symptoms (Int-Veen et al., 2023). Moreover, Rosenbaum et al. (2017) found that trait rumination had a stronger and more widespread impact on resting-state FC than state rumination and was negatively correlated with FC in the cortical regions of the DMN. Wei et al. (2024) replicated these findings using advanced connectome-based predictive modeling. Their results further indicated both state and trait rumination show increased FC between the DMN and FPN. These findings suggest that specific prefrontal regions and respective changes in FC within the FPN and/or DMN may serve as neurobiological markers of rumination. Please note, however, that the direction of causality in ruminative thinking and the associated brain activation patterns are not yet established. This is well summarized by Nolen-Hoeksema et al. (2008): “Certain [ …] changes in neural activity may be both causes and consequences of rumination.” (Nolen-Hoeksema et al., 2008, p. 411).

In this context, Non-Invasive Brain Stimulation (NIBS) is a valuable tool by which cortical brain activity can be modulated and the causal involvement of brain regions in the aforementioned processes can be investigated. What is particularly interesting in this context is the preliminary findings of Liston et al. (2014). Applying rTMS to the left DLPFC in 17 patients with depression, the authors found that a 5-week treatment normalized depression-related subgenual hyperconnectivity in the DMN. Interestingly, however, rTMS did not alter diminished connectivity within the FPN (Liston et al., 2014). Specifically investigating the effect of stimulation of the left DLPFC in the context of rumination, there are two studies using TBS, a variant of rTMS (De Smet et al., 2024; De Witte et al., 2020). Both of the aforementioned studies used the Trier Social Stress Test (TSST; Kirschbaum et al., 1993), a highly potent stressor that has repeatedly been found to elicit ruminative thinking (Gianferante et al., 2014; Hilt et al., 2015; Rosenbaum et al., 2021; Rosenbaum et al., 2018a; Shull et al., 2016; Zoccola et al., 2014). Both studies applied TBS to the left DLPFC and while De Smet et al. (2024) observed no general effect of TBS on post-stress state rumination, De Witte et al. (2020) found that higher trait rumination was partly associated with increased state rumination following the stressor but only following sham and not following two consecutive sessions of excitatory intermittent TBS (iTBS). Unfortunately, a key limitation of both studies is the lack of neural data assessment, as the effects of TBS are inferred solely from behavioral or physiological measures. As a result, the absence of observable TBS effects at these levels cannot be conclusively linked to a lack of neural effects.

In this study, we aimed to extend the findings of De Smet et al. (2024) and De Witte et al. (2020) by assessing respective changes in brain activation using functional Near-Infrared Spectroscopy (fNIRS). Specifically, we wanted to examine changes in resting-state FC before and after TBS applied to the left DLPFC, as well as following the stress induction via the TSST. Furthermore, as the Ventrolateral Prefrontal Cortex (VLPFC) “may be the best-supported target to affect stress modulation of emotional responses” (Moses et al., 2023), we conducted a second study with the exact same experimental design to ensure comparability but used the right VLPFC as a stimulation target in order to investigate the effect of contralateral stimulation. Prior studies have primarily focused on stimulation of the left DLPFC for several reasons. First, from a methodological standpoint, the left DLPFC is one of the most commonly targeted regions in NIBS research (Lefaucheur et al., 2014), particularly in studies on depression and cognitive control, which provides a strong empirical foundation for its use. Conceptually, the left DLPFC has been consistently associated with top-down regulation of emotion and thought processes (Ochsner et al., 2012), including the modulation of rumination (Kühn et al., 2014; Rosenbaum et al., 2018a,b,c, 2021). In contrast, the right VLPFC, while also implicated in emotional regulation and inhibitory control (Aron et al., 2004; Buhle et al., 2014), presents greater challenges in terms of precise targeting due to its more ventral and lateral anatomical location. This has likely contributed to its less frequent use in stimulation studies. The relative scarcity of stimulation studies focusing on this region highlights a notable gap in the literature, warranting further investigation into its potential for neuromodulatory interventions. Given these considerations, this study aimed to directly compare stimulation over the left DLPFC with stimulation over the right VLPFC to better understand their potentially distinct roles in stress-related rumination. To ensure high statistical power, we employed a between-subject design in which 88 participants, comprising high and low trait ruminators, attended two laboratory appointments with identical experimental setups. Participants received either active stimulation (cTBS or iTBS) or sham TBS (sTBS), with stimulation and appointment order randomized across groups and conditions. We hypothesized that iTBS would increase FC, particularly in the stimulation target and the associated FPN, while the presumably inhibitory cTBS would lead to decreased connectivity during the resting-state measurement following TBS and the stress induction.

## 2 Methods

### 2.1 Sample

For each study, following an a priori power analysis, we aimed to recruit a total of 88 right-handed healthy volunteers aged 18–50 years. Participants were recruited by means of circular emails via the university mailing list (for a list of inclusion and exclusion criteria of both studies, see supplementary material S1, for CONSORT diagrams of both studies, see supplementary material S2). The samples each comprised 44 low and 44 high trait ruminators, who were screened using the Ruminative Response Scale (RRS; Treynor and Gonzalez, 2003) and further assigned to receive either cTBS (*n* = 44) or iTBS (*n* = 44). Please note that each stimulation condition included 22 low and 22 high ruminators^1^. The studies each involved two laboratory appointments, scheduled at least 5 weeks apart. During these appointments, participants received either active (cTBS or iTBS) or sham stimulation (sTBS). The order in which participants received each type of stimulation was randomized and balanced across stimulation groups.

### 2.2 Experiment

**Fig. 1:**
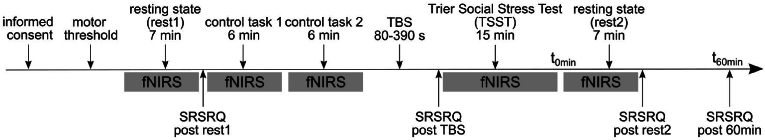
Overview of the experimental procedure. For more details on the control tasks and hemodynamic changes due to the TSST, we refer to Int-Veen et al. (2025). SRSRQ = Stress-Reactive State Rumination Questionnaire, TBS = Theta Burst Stimulation, TSST = Trier Social Stress Test. Motor threshold was determined over C3 (study 1) and C4 (study 2); TBS was applied to the left DLPFC (F3) in study 1 and the right VLPFC (F8) in study 2.

All participants provided written informed consent prior to participating. The procedure was the same at both appointments, except for the type of stimulation participants received. Upon arrival at the laboratory, each participant's resting motor threshold was determined. Next, participants were prepared for the fNIRS measurement and given instructions about the upcoming 7-minute resting-state measurement (rest1). They were asked to let their mind wander while being seated, keep their eyes open, and avoid excessive movement. A friendly study nurse was present in the same room throughout this time. After rest1, participants completed the Stress-Reactive State Rumination Questionnaire (SRSRQ; Int-Veen, Laicher et al., n.d.) as a behavioral measure of state rumination. Afterward, participants completed two control tasks, each lasting 6 min. In these tasks, they were instructed to either read numbers aloud or perform mental arithmetic similar to the TSST arithmetic task, but without time constraints or social pressure. Then, participants received TBS, followed by the assessment of another SRSRQ. Finally, participants underwent the Trier Social Stress Test (TSST; Kirschbaum et al., 1993). For details on the control tasks and TSST we refer to the respective publications on the task-based neural activation under stress investigating the hemodynamic changes during the aforementioned tasks (study 1/left DLPFC: Int-Veen et al., 2025; study 2/right VLPFC: Int-Veen, et al., (n.d.)) as well as supplementary material S3. In this paper, we aimed to analyze changes in resting-state functional connectivity during rest1 and rest2. Directly following the TSST, another 7-min resting state analogue to the first one was conducted (rest2) and another SRSRQ was assessed (SRSRQ post rest2). Finally, participants rested for 60 min after the TSST to recover, before completing the final SRSRQ. After both appointments were completed, participants were debriefed and received either monetary compensation (100 €) or course credit (6 h). This study was approved by the ethics committee at the University Hospital and University of Tübingen (673/2019BO1). For an overview of the experimental procedure, see Fig. 1.

**TBS.** Both the determination of the resting motor threshold and the neurostimulation were conducted in a separate room by a study nurse who was not otherwise involved in the experiment. We used a MagVenture MagPro X100 Stimulator (MagVenture, Farum, Denmark) and a figure-eight shaped coil (MagVenture C-B60) to determine the motor threshold. In study 1, we used the relative frequency method (Rothwell et al., 1999) by positioning the coil approximately over C3, and gradually reducing the intensity of the magnetic pulses until a visible muscle twitch occurred in the right thumb in fewer than 50 % of single pulses. In study 2, motor threshold was determined over C4 with an electromyogram using a 4-channel EMG-EP-system (Schreiber & Tholen Medizintechnik GmbH, Stade; EMG-EP = electromyography (EMG) and evoked potential (EP) system), a device used to record muscle activity and evoked potentials for neurophysiological assessment. After sanitizing the corresponding skin areas, two gold-plated surface cup electrodes of 11 mm diameter were placed on the right abductor pollicis brevis muscle (one thenar and one on the proximal phalanx) and one reference electrode on the inner arm below the wrist. Stimulation intensity was reduced until the amplitude of motor-evoked potentials of 50 μV in less than 50 % of 10 consecutive stimuli was recorded. For the neurostimulation, the coil was placed over the left DLPFC (study 1) or over the right VLPFC (study 2) at 80 % of the resting motor threshold. After disinfecting the skin, two pre-gelled surface electrodes (28 × 20 mm) were placed 1 cm around the stimulation site. A low current was applied via these electrodes to create a superficial sensation during active and sham stimulation in order to help maintain participant blinding. There were no significant differences in stimulation intensities between TBS conditions (cTBS, iTBS and sTBS) or between the studies. Participants received standardized written instructions about the neurostimulation (see supplementary material S4). The stimulation was performed while participants were seated in an EEG chair. A figure-eight shaped coil with active cooling (MagVenture Cool-B65 Active/Placebo coil) was positioned over F3 (study 1) or F8 (study 2). Participants were randomly assigned to receive either cTBS or iTBS. cTBS consisted of a single 80-s train of uninterrupted TBS, delivering 400 bursts (each containing 3 pulses at 50 Hz) at a burst frequency of 5 Hz. iTBS was applied in 40 cycles, each containing a 2-s theta burst train (10 bursts of 3 pulses each), followed by an 8-s rest period, totaling 390 s (Huang et al., 2005). For both protocols, the stimulation parameters included a total of 1200 pulses. Each participant received active stimulation at one appointment and sham stimulation of the same duration at the other. The order of active and sham stimulation was randomized and balanced across groups. The Cool-B65 Active/Placebo coil allowed for double-blind stimulation, automatically switching between active and sham TBS by flipping the coil. Overall, blinding was successful in study 1: A binomial test revealed that participants, when asked to guess whether they had received real or sham stimulation, did not identify the stimulation type significantly above chance level (*n* = 161; correct identifications = 92; *p* = 0.083, 95 % CI [0.35, 0.51]). When analyzed by appointment, blinding was effective at the first appointment (*n* = 77; correct = 38; *p* = 0.999, 95 % CI [0.39, 0.62]). However, at the second appointment, participants were able to identify the stimulation type significantly above chance, (*n* = 84; correct = 54; *p* = 0.012, 95 % CI [0.26, 0.47]). In study 2, blinding was not successful overall (*n* = 175; correct = 104; *p* = 0.015, 95 % CI [0.33, 0.48]). When analyzed by appointment, participants were successfully blinded during the first (*n* = 88; correct = 40; *p* = 0.456, 95 % CI [0.44, 0.65]), but not during the second appointment (*n* = 87; correct = 64; *p* < 0.001, 95 % CI [0.18, 0.37]). Please note that the discrepancy in participant numbers between measurement time points 1 and 2 is attributable to instances where the relevant question was either left unanswered or answered ambiguously.

**Fig. 2:**
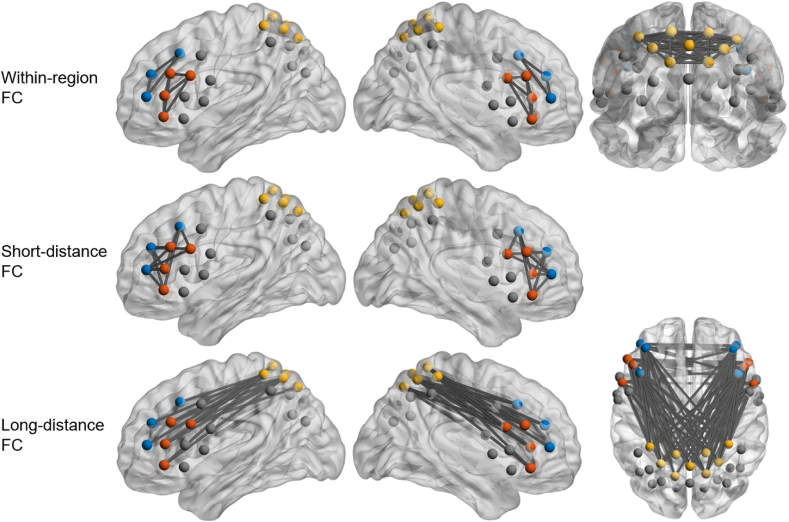
Definition of regions of interest in the analysis and the corresponding within, short-distance and long-distance FC measures. The brain networks were visualized with the BrainNet Viewer (Xia et al., 2013). As a brain surface template we used the ICBM152 (MNI/Talairach) template. Dots represent fNIRS channels. Orange represents the bilateral Ventrolateral Prefrontal Cortex, blue represents the bilateral Dorsolateral Prefrontal Cortex, yellow represents the Somatosensory Association Cortex. Please note that for the analysis, we calculated the mean FC, averaging the connectivity for channels of respective ROIs (e.g. for long-distance FC between the DLPFC and SAC, the mean was calculated for FC values between the six blue channels (DLPFC) and the nine yellow channels (SAC)).

**fNIRS.** Relative changes in cortical oxygenation were assessed during both resting-states using an ETG-4000 Optical Topography System with a sampling rate of 10 Hz (continuous wave multichannel fNIRS; Hitachi Medical Co., Japan). We assessed a total of 46 channels separated on two frontal probesets (12 channels, i.e. 5 emitters and 4 detectors each) covering the bilateral Dorsolateral (DLPFC) and Ventrolateral Prefrontal Cortex (VLPFC) as well as one parietal probeset (22 channels, i.e. 8 emitters and 7 detectors) covering the Somatosensory Association Cortex (SAC). Emitters were semiconductor lasers and detectors were avalanche photodiodes. Light was emitted at two wavelengths (695 ± 20 nm and 830 ± 20 nm) with 2.0 ± 0.4 mW for each wavelength at each optode. Optodes were integrated into EEG caps with 3 cm inter-optode distances and placed with reference to Fpz and Cz. Scalp-brain correspondence was estimated based on Okamoto et al. (2004), Okamoto and Dan (2005) and Singh et al. (2005). Relative changes in oxygenated and deoxygenated hemoglobin were calculated by means of the modified Beer-Lambert Law (Sassaroli and Fantini, 2004) using custom MATLAB 2024 scripts. Preprocessing included interpolation of single noisy channels, correction of motion artifacts using Temporal Derivative Distribution Repair (Fishburn et al., 2019), Correlation-Based Signal Improvement (Cui et al., 2010) and bandpass filtering to remove low-frequency baseline drifts (< 0.01 Hz) and high-frequency noise (> 0.1 Hz). To remove artifacts induced due to data correction, additional channel interpolation was performed, followed by a global signal reduction with a spatial Gaussian kernel filter (σ = 40) and z-standardization of the signal. As FC measures, we computed Pearson correlation coefficients for each ROI (left and right DLPFC, left and right VLPFC and SAC) with each ROI as well as within each ROI. As in Zhu et al. (2017) we separated these connections into within-region FC (within each region), short-distance FC (between the ipsilateral DLPFC and VLPFC) and long-distance FC (between contralateral DLPFC, VLPFC and SAC) (see Fig. 2). Brain Net figures were plotted using BrainNet Viewer (Xia et al., 2013).

**Data plan.** Data analysis was performed using R version 4.4.1 and RStudio Version 2024.12.1.563 (RStudio Team, 2024) with the packages lme4, lmerTest and ggplot2. Multivariate outliers were identified using Mahalanobis distances, which were computed based on the covariance matrix. Data points exceeding the critical chi-square threshold for *p* < 0.001 were classified as outliers.

For the behavioral analysis of state rumination, we fitted a linear mixed model with time (four assessment points), group (low vs. high ruminators), and condition (sTBS vs. cTBS vs. iTBS) as fixed effects. To account for individual variability and the two consecutive appointments, we included random intercepts for subjects and a random interaction term between subject and appointment. No random slopes were included. The model structure was consequently as follows: lmer(staterum ∼ time ∗ group ∗ condition + (1 | subject) + (1 | subject:appointment), data = dat). This analysis has already been reported in previous publications detailing the hemodynamic changes observed in these studies (Int-Veen et al., 2025; Int-Veen, Laicher et al., n.d. however, it is presented here again for completeness.

Next, we analyzed the fNIRS FC data: We fitted separate linear mixed models for each FC measure. Each linear mixed model examined the effects of time (rest1 vs. rest2), group (low vs. high ruminators) and condition (sTBS vs. cTBS vs. iTBS) on FC, with random intercepts for subject and the interaction between subject and appointment to account for individual subject-level variability and the two consecutive appointments. Lastly, we explored the relationship between behavioral and neural data by examining the correlation between state rumination ratings post rest2 and FC increases, calculated as the change in FC from rest1 to rest2. This exploratory analysis was not corrected for multiple comparisons.

For the behavioral analysis as well as the analysis of the fNIRS FC data, we report Type III Wald chi-squared statistics for the fixed effects. In case of significant three-way interaction effects (time∗group∗condition), we fitted separate models depending on group prior to conducting post-hoc tests using pairwise comparisons of estimated marginal means with the emmeans package (Lenth & others, 2022) and using the Tukey method to adjust for multiple comparisons for the behavioral analysis as well as the analysis of the fNIRS FC data.

In cases where a significant three-way interaction between time, group, and condition emerged, we fitted separate models for each group (i.e., low and high ruminators) and subsequently conducted post-hoc tests. For fNIRS FC data, we conducted post-hoc tests to examine the differences between time points (rest1 vs. rest2) separately for each TBS condition. Additionally, we analyzed the differences between TBS conditions (sTBS - cTBS, sTBS - iTBS and cTBS - iTBS) at each time point as well as differences between TBS conditions in the change from rest1 to rest2 (rest2 - rest1 for sTBS vs. rest2 - rest1 for iTBS vs. rest2 - rest1 for cTBS).

When a significant time ∗ condition interaction was observed, we proceeded directly with the same post-hoc comparisons as described above. In cases where a significant time ∗ group interaction emerged — this applied only to the behavioral analysis of state rumination — we performed post-hoc tests to examine (1) differences between time points (rest1 vs. rest2) separately for low and high ruminators and (2) group differences at each time point.

Prior to the main analyses, we examined potential baseline differences by fitting the respective models to data from the first measurement time point only, assuming a random intercept for each participant. In the behavioral data, we observed significantly higher levels of state rumination after rest1; that is, prior to stimulation, in both studies (see Behavioral Results). In contrast, the analysis of fNIRS FC data revealed no significant baseline differences between groups or stimulation conditions.

In the main figures, we have illustrated the highest-order significant interactions for clarity. For interested readers, additional figures are provided in supplementary material S5, presenting the results separately for low and high ruminators for all main figures where such subgroup distinctions were not originally shown.

For the sake of clarity, the results of the two studies will first be reported separately before being jointly discussed in the subsequent section.

## 3 Results study 1: TBS applied to the left DLPFC

### 3.1 Behavioral Results - study 1

**Fig. 3:**
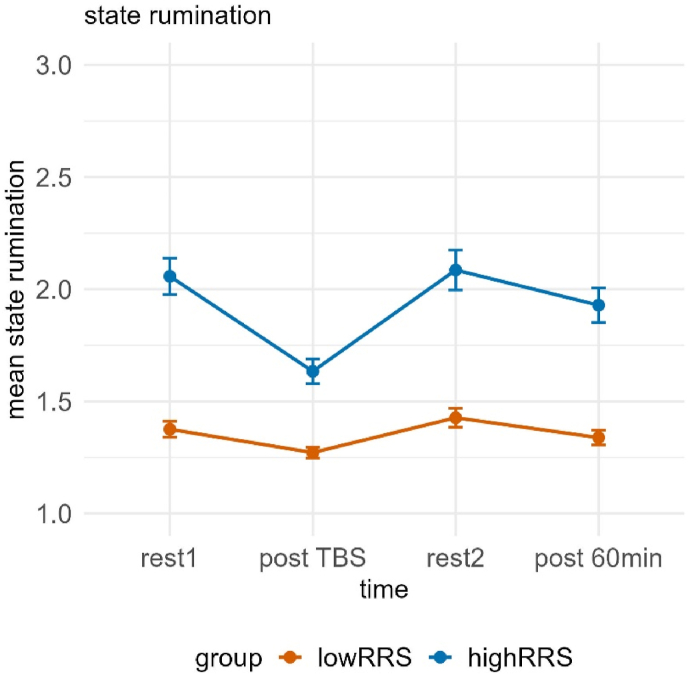
Mean state rumination ratings dependent on time and group (low vs. high ruminators). Error bars represent ±1 standard error of the mean. Post-hoc tests revealed significant group differences at all time points. Low ruminators showed an increase post TBS to rest2, while high ruminators exhibited a significant decrease from rest1 to post TBS, followed by significant increases at rest2 and from post TBS to post 60 min. rest1 = resting-state measurement 1, TBS = Theta Burst Stimulation, rest2 = resting-state measurement 2, post 60min = 60 min after the TSST.

The mixed model investigating state rumination ratings yielded a significant interaction of time and group, $\chi^{2}$(3) = 12.720, *p* < 0.01, but no impact of the stimulation in the form of a significant interaction of group and condition or main effect of condition (all *p*'s > 0.156). Post-hoc tests of the interaction revealed significant differences between low and high ruminators at all time points (all *p*'s < 0.01). Low ruminators showed only an increase in state rumination from the stimulation (post TBS) (*M* = 1.27, *SE* = 0.07) to post rest2 (*M* = 1.44, *SE* = 0.07) (i.e. due to the stress induction), *t*(472) = −3.075, *p* < 0.05, *d* = −0.494, whereas high ruminators exhibited a significant decrease from rest1 (*M* = 2.09, *SE* = 0.07) to post TBS (*M* = 1.66, *SE* = 0.07), *t*(471) = 7.355, *p* < 0.001, *d* = 1.251, significant increases after the TSST during rest2 (*M* = 2.10, *SE* = 0.07), *t*(471) = −7.463, *p* < 0.001, *d* = −1.270, as well as significant increases from TBS to 60 min post TSST (*M* = 1.98, *SE* = 0.07), *t*(471) = −5.422, *p* < 0.001, *d* = 0.924 (see Fig. 3).

### 3.2 Neural results - study 1

#### 3.2.1 Short-distance FC - study 1

**Fig. 4:**
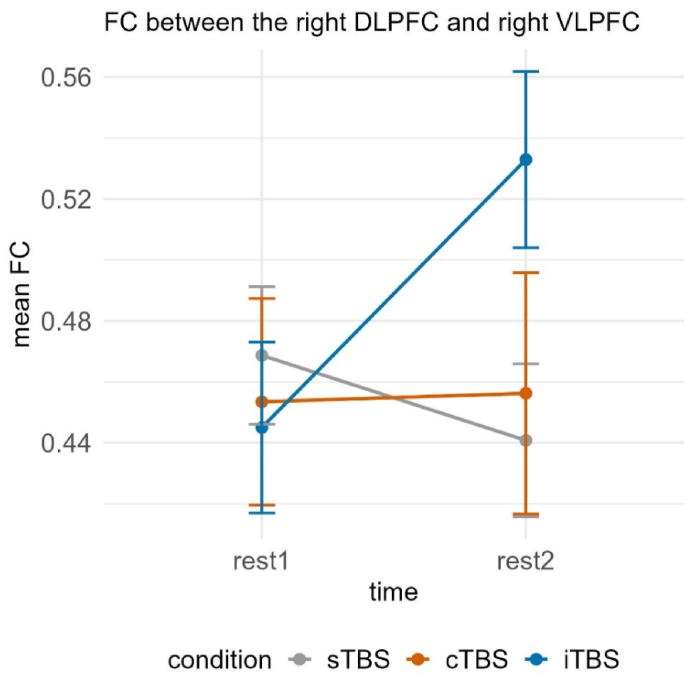
Functional connectivity between the right DLPFC and right VLPFC dependent on time (rest1 vs. rest2) and condition (sTBS vs. cTBS vs. iTBS). Error bars represent ±1 standard error of the mean. Post-hoc tests revealed significant increases in FC between rest1 and rest2 following iTBS and further a significant difference between iTBS and sTBS at rest2. sTBS = sham Theta Burst Stimulation, cTBS = continuous Theta Burst Stimulation, iTBS = intermittent Theta Burst Stimulation.

Investigating short-distance FC, we observed a significant interaction between time and condition for FC between the right DLPFC and right VLPFC, $\chi^{2}$(2) = 9.280, *p* < 0.01. Post-hoc tests yielded significant increases in FC between rest1 (*M* = 0.448, *SE* = 0.032) and rest2 (*M* = 0.532, *SE* = 0.032) following iTBS, *t*(157) = −2.202, *p* < 0.05, *d* = −0.488, and consequently significantly higher FC during rest2 following iTBS (*M* = 0.532, *SE* = 0.032) compared to sTBS (*M* = 0.441, *SE* = 0.024), *t*(225) = −2.597, *p* < 0.05, *d* = −0.525. We further observed significant differences in the change from rest1 to rest2 between sTBS (*M* = −0.028, *SE* = 0.027) and iTBS (*M* = 0.087, *SE* = 0.039), *t*(108) = −2.527, *p* < 0.05, *d* = −0.497 (see Fig. 4).

#### 3.2.2 Long-distance FC - study 1

**Fig. 5:**
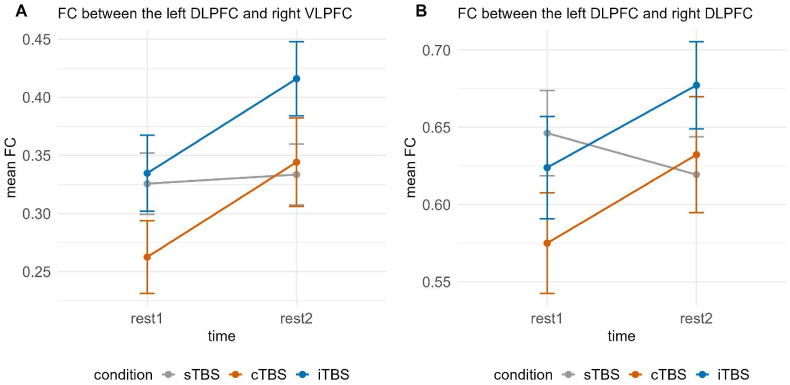
Functional connectivity between the left DLPFC and right VLPFC (A) as well as between the left DLPFC and right DLPFC (B) dependent on time (rest1 vs. rest2) and condition (sTBS vs. cTBS vs. iTBS). Error bars represent ±1 standard error of the mean. Post-hoc tests indicated significant increases in FC between rest1 and rest2 only following cTBS and significant increases following iTBS in case of FC between the left DLPFC and right VLPFC (A). sTBS = sham Theta Burst Stimulation, cTBS = continuous Theta Burst Stimulation, iTBS = intermittent Theta Burst Stimulation.

Investigating long-distance FC, there was a significant interaction between time and condition for FC between the left DLPFC and right VLPFC, $\chi^{2}$(2) = 6.379, *p* < 0.05. Post-hoc tests showed significant increases in FC between rest1 (*M* = 0.272, *SE* = 0.035) and rest2 (*M* = 0.354, *SE* = 0.035) following cTBS, *t*(157) = −2.001, *p* < 0.05, *d =* −0.448 and also marginally significant increases between rest1 (*M* = 0.328, *SE* = 0.034) and rest2 (*M* = 0.406, *SE* = 0.034) following iTBS, *t*(157) = −1.915, *p* = 0.057, *d* = −0.424 (see Fig. 5A).

We further observed a significant interaction between time and condition, $\chi^{2}$(2) = 6.118, *p* < 0.05, as well as a significant interaction between time and group, for the functional connectivity between the left and right DLPFC, $\chi^{2}$(1) = 6.293, *p* < 0.05. Post-hoc tests of the interaction between time and condition yielded no significant differences between rest1 and rest2 for either condition and no significant differences between conditions at respective time points (all *p*'s > 0.173) (see Fig. 5B).

**Fig. 6:**
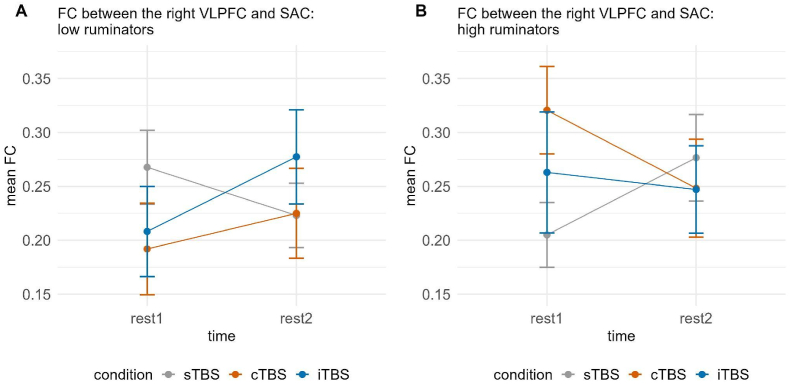
Functional connectivity between the right VLPFC and SAC dependent on time (rest1 vs. rest2) and condition (sTBS vs. cTBS vs. iTBS) for low (A) and high ruminators (B). Error bars represent ±1 standard error of the mean. Post-hoc tests did not yield significant results, either before or after correction for multiple comparisons. sTBS = sham Theta Burst Stimulation, cTBS = continuous Theta Burst Stimulation, iTBS = intermittent Theta Burst Stimulation.

A significant three-way interaction of time, group and condition emerged for FC between the right VLPFC and SAC, $\chi^{2}$(2) = 8.246, *p* < 0.05; however, fitting separate models depending on the group only yielded marginally significant interactions of time and condition in the case of both groups (all *p*'s > 0.094), and all post-hoc tests did not yield significance (all *p*'s > 0.072). This was also the case when we did not correct for multiple comparisons (see Fig. 6).

Lastly, we observed a significant main effect of time in case of FC between the right DLPFC and left VLPFC, $\chi^{2}$(1) = 7.788, *p* < 0.01, reflecting overall significant increases in FC over time.

#### 3.2.3 Within FC - study 1

**Fig. 7:**
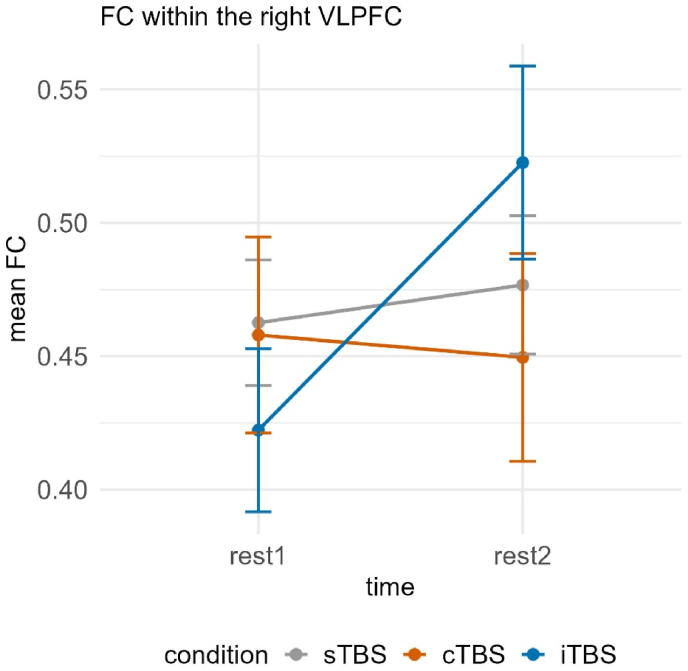
Functional connectivity within the right VLPFC dependent on time (rest1 vs. rest2) and condition (sTBS vs. cTBS vs. iTBS). Error bars represent ±1 standard error of the mean. Post-hoc tests revealed significant increases in FC between rest1 and rest2 following iTBS. sTBS = sham Theta Burst Stimulation, cTBS = continuous Theta Burst Stimulation, iTBS = intermittent Theta Burst Stimulation.

Regarding FC within the right VLPFC, there was a significant interaction of time and condition, $\chi^{2}$(2) = 9.109, *p* < 0.05. Post-hoc tests revealed significant increases in FC between rest1 (*M* = 0.427, *SE* = 0.035) and rest2 (*M* = 0.523, *SE* = 0.035) following iTBS, *t*(157) = −2.260, *p* < 0.05, *d* = −0.500 (see Fig. 7).

#### 3.2.4 Exploratory analysis - study 1

The correlation of FC increases with state rumination ratings only yielded significance in case of FC within the left VLPFC, *r*(167) = 0.161, *p* < 0.05, indicating higher state rumination ratings following the stress induction (rest2) in the case of higher increases in FC within the left VLPFC (all other *p*'s > 0.149).

## 4 Results study 2 TBS applied to the right VLPFC

### 4.1 Behavioral Results - study 2

Analogue to the results of study 1, we observed a significant interaction of time and group, $\chi^{2}$(3) = 8.451, *p* < 0.05. Post-hoc tests of the interaction revealed significant differences between low and high ruminators at all time points (all *p*'s < 0.001). There was no significant interaction of group and condition or main effect of condition.

**Fig. 8:**
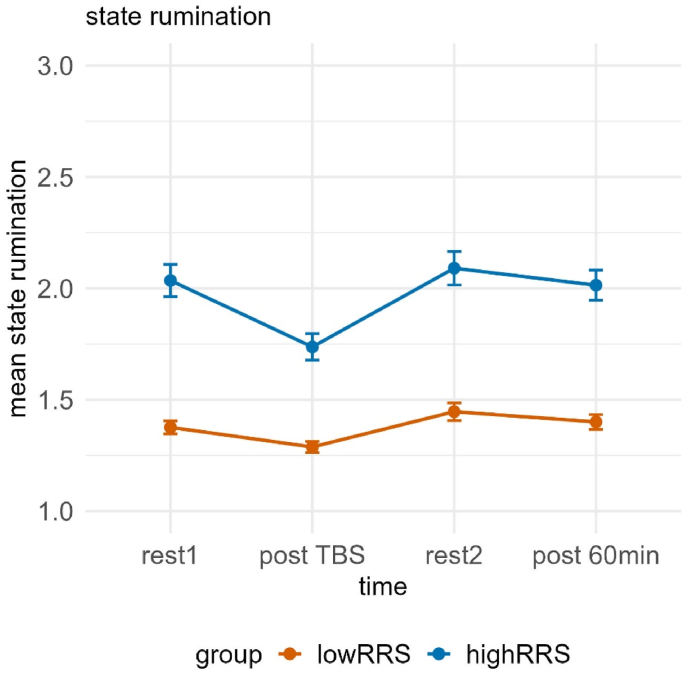
Mean state rumination ratings dependent on time and group (low vs. high ruminators). Error bars represent ±1 standard error of the mean. Post-hoc tests revealed significant group differences at all time points. Low ruminators showed an increase post TBS to rest2, while high ruminators exhibited a significant decrease from rest1 to post TBS, followed by significant increases at rest2 and from post TBS to post 60 min. rest1 = resting-state measurement 1, TBS = Theta Burst Stimulation, rest2 = resting-state measurement 2, post 60min = 60 min after the TSST.

Low ruminators showed only an increase in state rumination from TBS (*M* = 1.31, *SE* = 0.07) to rest2 (*M* = 1.46, *SE* = 0.07) (i.e. due to the stress induction), *t*(488) = −3.171, *p* < 0.01, *d* = −0.509, whereas high ruminators exhibited a significant decrease from rest1 (*M* = 2.09, *SE* = 0.07) to TBS (*M* = 1.79, *SE* = 0.07), *t*(487) = 6.415, *p* < 0.001, *d* = 1.056, followed by a significant increase from TBS to rest2 (*M* = 2.12, *SE* = 0.07) (i.e. due to the stress induction), *t*(487) = −6.906, *p* < 0.001, *d* = 1.137, as well as significant increases from TBS to 60 min post TSST (*M* = 2.05, *SE* = 0.07), *t*(487) = −5.471, *p* < 0.001, *d* = 0.901 (see Fig. 8).

## 5 Neural results - study 2

### 5.1 Short-distance FC - study 2

Investigating short-distance FC, we observed no significant effects (all *p*'s > 0.253).

### 5.2 Long-distance FC - study 2

**Fig. 9:**
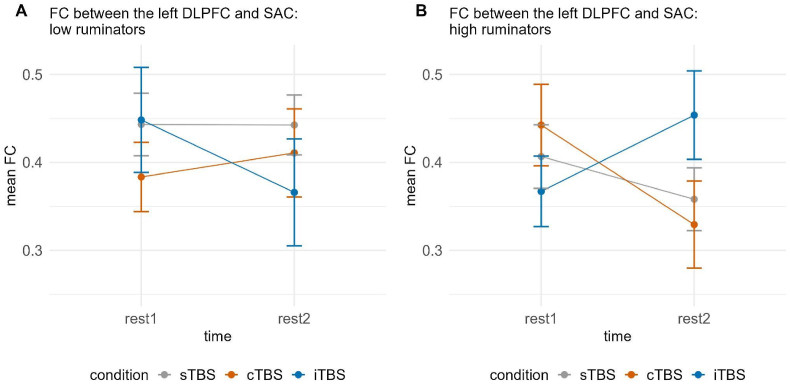
Functional connectivity between the left DLPFC and SAC dependent on time (rest1 vs. rest2) and condition (sTBS vs. cTBS vs. iTBS) for low (A) and high ruminators (B). Error bars represent ±1 standard error of the mean. Post-hoc tests only indicated marginally significant decreases between rest1 and rest2 following cTBS in high ruminators (B). sTBS = sham Theta Burst Stimulation, cTBS = continuous Theta Burst Stimulation, iTBS = intermittent Theta Burst Stimulation.

For long-distance FC, a significant three-way interaction of time, group and condition for FC between the left DLPFC and SAC emerged, $\chi^{2}$(2) = 7.230, *p* < 0.05. Fitting separate models dependent on the group yielded only a marginally significant interaction of time and condition in high ruminators, $\chi^{2}$(2) = 5.275, *p* = 0.072. Post-hoc tests also only indicated marginally significant decreases between rest1 (*M* = 0.439, *SE* = 0.048) and rest2 (*M* = 0.326, *SE* = 0.048) following cTBS, *t*(78) = 1.845, *d* = 0.569 (see Fig. 9).

We further observed a significant main effect of time in case of FC between the left and right DLPFC, $\chi^{2}$(1) = 4.096, *p* < 0.05, reflecting overall significant increases in FC over time.

### 5.3 Within FC - study 2

**Fig. 10:**
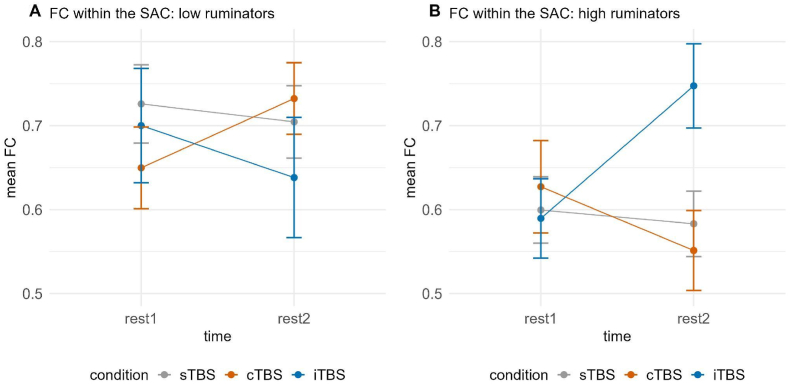
Functional connectivity within the SAC dependent on time (rest1 vs. rest2) and condition (sTBS vs. cTBS vs. iTBS) for low (A) and high ruminators (B). Error bars represent ±1 standard error of the mean. Post-hoc tests revealed significant increases in FC from rest1 to rest2 following iTBS, as well as significantly higher FC during rest2 after iTBS compared to both cTBS and sTBS in high ruminators (B). sTBS = sham Theta Burst Stimulation, cTBS = continuous Theta Burst Stimulation, iTBS = intermittent Theta Burst Stimulation.

There was a significant three-way interaction of time, group and condition for FC within the SAC, $\chi^{2}$(2) = 11.662, *p* < 0.01. Fitting separate models dependent on group revealed a significant interaction of time and condition only in high ruminators, $\chi^{2}$(2) = 9.869, *p* < 0.01. Post-hoc tests indicated significant increases in FC between rest1 and rest2 following iTBS, *t*(78) = −2.792, *p* < 0.01, *d* = −0.906 and consequently significantly higher FC during rest2 following iTBS (*M* = 0.562, *SE* = 0.051) compared to cTBS (*M* = 0.641, *SE* = 0.049), *t*(110) = −2.384, *p* < 0.05, *d =* −0.885, and following iTBS compared to sTBS (*M* = 0.610, *SE* = 0.037), *t*(102) = −2.454, *p* < 0.05, *d* = −0.723 (see Fig. 10).

Lastly, we observed a significant main effect of time in case of FC within the left DLPFC, $\chi^{2}$(1) = 4.013, *p* < 0.05, reflecting overall significant increases in FC over time.

### 5.4 Exploratory analysis - study 2

The correlation of FC increases with state rumination ratings only yielded significance regarding FC within the right DLPFC, *r*(173) = 0.181, *p* < 0.05, indicating higher state rumination ratings following the stress induction (rest2) in the case of higher increases in FC within the right DLPFC (all other *p*'s > 0.276).

## 6 Discussion

This study aimed to investigate the neuromodulatory effect of Theta Burst Stimulation (TBS) on changes in functional connectivity (FC) due to an experimental stress induction using the Trier Social Stress Test (TSST; Kirschbaum et al., 1993). Using a between-subjects design where low and high ruminators either received cTBS or iTBS compared to sTBS, we conducted two studies with different stimulation targets: In the first study, we applied TBS to the left DLPFC as a prominent target of the Fronto-Parietal Network (FPN), while the second study targeted the contralateral right VLPFC (see Moses et al., 2023).

In both studies, our behavioral analyses of state rumination ratings indicated that the TSST successfully induced state rumination, with a stronger effect in high compared to low trait ruminators, as expected. However, no direct effect of stimulation was observed at the behavioral level. This is well in line with the findings of previous studies not observing a general effect of TBS on state rumination (De Smet et al., 2024; De Witte et al., 2020) and further stresses the importance of the assessment of neural data in this context so that the absence of observable TBS effects at behavioral levels can be linked to a lack of neural effects.

Upon analyzing the neural data, we observed consistent and significant increases in FC in response to iTBS. In contrast, the effects of cTBS were generally either comparable to those of sTBS or similar to iTBS, in that they also demonstrated an enhancing effect on the synchronization of brain regions. For instance, in study 1, we found increased FC between the right DLPFC and right VLPFC, as well as within the right VLPFC following iTBS compared to sTBS (and cTBS), respectively. However, no significant changes in FC over time were observed for cTBS or sTBS. Notably, the stimulation had an effect on the contralateral hemisphere, as stimulation of the left DLPFC resulted in altered connectivity in the right hemisphere. Given the strong interconnectivity between these regions, this finding aligns with previous evidence that TMS not only influences the activity of the directly stimulated neurons but also modulates functionally connected brain areas (Gratton et al., 2013; Ilmoniemi et al., 1997; Komssi et al., 2002). However, research on this phenomenon remains limited. In contrast to the consistent pattern in which iTBS led to increased FC, while cTBS and sTBS showed no significant changes in FC, both iTBS and cTBS exhibited enhancing effects on interhemispheric synchronization. This suggests that active stimulation—regardless of its assumed excitatory or inhibitory effects on neuronal activity—modulated functional connectivity between the left and right hemispheres. Specifically, we observed significant increases in FC between the left DLPFC and right VLPFC, as well as between the left DLPFC and right DLPFC, following both cTBS and iTBS. However, the increase in FC between the left DLPFC and right VLPFC was only marginally significant for iTBS. No changes in FC were observed following sTBS. It is important to note that these findings pertain exclusively to study 1, in which stimulation was applied to the left DLPFC. In study 2, where stimulation targeted the right VLPFC, fewer brain regions exhibited a significant effect of TBS. This may be because the left DLPFC is an established critical target for investigating the FPN. In contrast, the right VLPFC is more strongly associated with stress modulation of emotional responses and cognitive reappraisal (Moses et al., 2023). Potentially, here, subcortical areas that are not measurable with fNIRS are primarily implicated. As a result, the right VLPFC may play a lesser role in FPN-associated processes, leading to its lower recruitment following the TSST.

Our mixed findings regarding the exact direction of cTBS effects align well with the findings of a recent meta-analysis by Kirkovski et al. (2023) that found that both inhibitory cTBS and excitatory iTBS over the prefrontal cortex can induce excitatory or inhibitory responses, depending on various factors. Recent reviews suggest that the limited intrasubject reliability and substantial intersubject variability observed in NIBS findings may be largely attributed to a failure to account for state-dependency — specifically, the cognitive brain state during stimulation (including cognitive, emotional, and perceptual states during different tasks performed), the spontaneous moment-to-moment fluctuations in oscillatory brain activity during stimulation (i.e. the corresponding neural state), and the neural activity prior to the stimulation (Bradley et al., 2022; Sack et al., 2024). Interestingly, Kirkovski et al. (2023) further state that “This apparent inconsistency in response to TBS effects, therefore, appears to go beyond task- or state-dependence when the PFC is targeted.” (Kirkovski et al., 2023). Along with methodological factors (e.g. variability in targeting approaches, coil placement) and the inherent complexity of neural organization of the frontal cortex, the authors argue that this variability most probably depends also on contextual factors such as the task demands. Others further argue that individual differences in psychological states may correlate with the magnitude and directionality of effects of NIBS (Schutter et al., 2023) and individual characteristics such as age or genetics have a substantial impact (Corp et al., 2020; Ridding and Ziemann, 2010). This emerging understanding challenges the traditional binary classification of TBS protocols as purely excitatory or inhibitory and underscores the need for further research (Goldsworthy et al., 2021; Hussain and Freedberg, 2025).

An important direction for future research might lie in further elucidating the precise relationship between TBS-induced effects on regional brain activity and alterations in FC. As previously noted, the latter has received comparatively less attention. Yet, in the context of compensatory network mechanisms, it is crucial to take these connectivity changes into account. Our analysis of hemodynamic responses within specific ROIs during the TSST revealed highly inconsistent and complex results (Int-Veen et al., 2025; Int-Veen et al. n.d.). On the one hand, the absence of detectable TBS effects on the neural activation under stress could be attributed to the strong prefrontal hemodynamic responses induced by the TSST, potentially masking any additional modulation through TBS. These effects may only become apparent during the subsequent resting-state period, once the task-evoked prefrontal hemodynamic response subsides. On the other hand, the discrepancy might reflect differences in the neural mechanisms targeted by TBS or the varying sensitivity of the respective analytic approaches. Further research is needed to disentangle these possibilities.

Interestingly, in both studies, we observed partly opposing effects of stimulation conditions in low and high ruminators. These effects were particularly evident in the FC between prefrontal regions in the hemisphere contralateral to the stimulated site and the SAC. Specifically, in study 1, following stimulation of the left DLPFC, we identified a three-way interaction of time, group, and stimulation condition in FC between the right VLPFC and SAC. Similarly, in study 2, where stimulation was applied to the right VLPFC, a comparable three-way interaction emerged in FC between the left DLPFC and SAC, as well as within the SAC. These three-way interactions appeared to be driven by differential effects of the stimulation conditions in high ruminators, with iTBS again exhibiting a predominantly enhancing effect on FC. In low ruminators, iTBS also sometimes led to an enhancing effect on FC; however, we also observed descriptive decreases in FC following iTBS, as well as similar effects across all three stimulation conditions. This, together with other studies showing a significant impact of cognitive or personality variables on TBS effects (Pulopulos et al., 2019; Schutter et al., 2023) and, interestingly, trait rumination (De Smet et al., 2024; De Witte et al., 2020), suggests a complex interplay of multiple factors in the inhibitory and excitatory effects of TBS also on FC. Further, our findings concerning interhemispheric effects are well in line with other studies finding generalized increases in network connectivity following rTMS using EEG and MRI (Gratton et al., 2013; Ilmoniemi et al., 1997; Komssi et al., 2002).

Nevertheless, a key limitation of the present studies must be acknowledged: in both samples, all participants were right-handed university students. This homogeneity restricts the external validity of the findings. In particular, factors such as age—which is known to significantly influence neural responses and outcomes in neuromodulation research—should be taken into account when interpreting the results. Another limitation is the compromised blinding in the case of study 2, particularly at the second appointment, where participants were able to identify the stimulation condition significantly above chance level. This lack of effective blinding introduces the possibility of expectancy effects or response biases that may have influenced the results. This may have limited the increase in state rumination, which in turn could account for the absence of significant TBS-related effects on behavioral outcomes. Although blinding was successful during the first session, the overall failure to maintain blinding across both sessions limits the internal validity of the findings and warrants cautious interpretation of the stimulation effects observed in study 2.

When examining the correlation between state rumination ratings following the TBS and TSST and changes in FC, we identified significant positive associations within the left VLPFC when stimulation was applied to the left DLPFC (study 1). Similarly, significant positive associations were observed within the right DLPFC when stimulation targeted the right VLPFC (study 2). However, it is important to interpret these correlations with caution, as they were not corrected for multiple comparisons. Given the involvement of the VLPFC and DLPFC in cognitive control and emotional regulation, the observed increased connectivity may suggest an effortful attempt to regulate or suppress ruminative thoughts following stress. These preliminary findings highlight the potential role of these regions in stress responses and the modulation of ruminative thought processes. In the absence of a TBS intervention, Rosenbaum et al. (2018a) reported similar findings: They found significant negative associations (not corrected for multiple comparisons) between increases in FC between the right DLPFC and Inferior Frontal Gyrus (IFG) and post-stress state rumination in a smaller but comparable sample of low and high trait ruminators. Their findings suggest that greater functional integration between the right DLPFC and IFG in response to the TSST was associated with lower post-stress rumination, which, together with the findings of the current studies, which underscores the relevance of the prefrontal regions in stress-reactive rumination.

While we observed significant effects of TBS at the neural level, specifically in terms of increased fronto-parietal functional connectivity, no corresponding impact was found at the behavioral level. One possible explanation is a lack of direct translation from neural changes to observable behavior. This could be due to neural effects not immediately manifesting in behavioral responses (after a single session of TBS), the changes in ruminative thinking being too subtle to detect (potentially reflecting a limitation in the sensitivity of the SRSRQ), or behavioral effects being masked by compensatory mechanisms at either the neural or behavioral level. For instance, increased cognitive effort and enhanced recruitment of prefrontal regions may have offset potential behavioral changes.

Future studies should consistently incorporate neural measurements to directly assess the effects of TBS, rather than solely inferring them from behavioral data. Additionally, it is crucial to consider the interactions between TBS effects and cognitive factors, as these relationships appear to be highly complex. This is particularly relevant in the context of rumination, where individual differences may significantly shape the impact of stimulation. A more comprehensive understanding of these dynamics will be essential for refining stimulation protocols and optimizing their effectiveness in both research and clinical applications. Taken together, these findings emphasize the value of neural assessments in TBS research and reveal how individual differences in rumination influence responses to stimulation. Exploring the link between TBS, fronto-parietal connectivity, and rumination could guide more personalized neuromodulation approaches.

## CRediT authorship contribution statement

**Isabell Int-Veen:** Writing – review & editing, Writing – original draft, Visualization, Software, Investigation, Formal analysis, Data curation. **Beatrix Barth:** Writing – original draft, Investigation. **Ramona Täglich:** Writing – original draft, Resources, Investigation. **Betti Schopp:** Writing – original draft, Resources, Investigation. **Hans-Christoph Nuerk:** Writing – original draft, Resources. **Christian Plewnia:** Writing – original draft, Resources. **Stefanie De Smet:** Writing – original draft. **Marie-Anne Vanderhasselt:** Writing – original draft, Validation, Supervision, Methodology. **Andreas J. Fallgatter:** Writing – original draft, Validation, Supervision, Resources. **Ann-Christine Ehlis:** Writing – original draft, Validation, Supervision, Resources, Project administration, Funding acquisition. **David Rosenbaum:** Writing – original draft, Validation, Supervision, Project administration, Funding acquisition, Conceptualization.

## Declaration of generative AI and AI-assisted technologies in the writing process

During the preparation of this work the authors used ChatGPT in order to assist with language refinement. After using this tool/service, the authors reviewed and edited the content as needed and took full responsibility for the content of the publication.

## Funding

This study was funded by the 10.13039/501100001659German Research Foundation (10.13039/501100001659DFG) (Project number RO 6676/1-1). SDS is funded by a FWO-10.13039/501100011878Flanders PhD fellowship (Grant Number: 3F001120). MAV received funding from FWO-10.13039/501100011878Flanders for research projects for fundamental research (Grant Numbers: G044222N; G044019N), and is supported by a grant for a Concerted Research Action of the Special Research Fund of 10.13039/501100004385Ghent University (01G00623).

## Declaration of competing interest

The authors declare the following financial interests/personal relationships which may be considered as potential competing interests: Isabell Int-Veen reports financial support was provided by 10.13039/501100001659Deutsche Forschungsgemeinschaft. Stefanie De Smet reports financial support was provided by FWO-10.13039/501100011878Flanders PhD fellowship. Marie-Anne Vanderhasselt reports financial support was provided by FWO-10.13039/501100011878Flanders for research projects for fundamental research. Marie-Anne Vanderhasselt reports financial support was provided by Concerted Research Action of the Special Research Fund of 10.13039/501100004385Ghent University. If there are other authors, they declare that they have no known competing financial interests or personal relationships that could have appeared to influence the work reported in this paper.

## Footnotes

1 Please note that in study 2 we accidently recruited one additional male high ruminator receiving sTBS at his first and iTBS at his second appointment.
