# Supplementary material for: Interhemispheric effects of iTBS on the fronto-parietal network: Evidence from dual-site stimulation
Source: Neurobiol Stress. 2025 Jul 10;37:100744. doi: 10.1016/j.ynstr.2025.100744 (PMC12284565; doi:10.1016/j.ynstr.2025.100744)
Supplement: Multimedia component 1 [file mmc1.pdf]

Supplementary material to

Interhemispheric Effects of iTBS on the Fronto-Parietal Network: Evidence from Dual-Site Stimulation

Isabell Int-Veen[1], Beatrix Barth[1,2,3], Ramona Täglich[1], Betti Schopp[1], Hans-Christoph Nuerk[2,4], Christian Plewnia[1,2], Stefanie De Smet[5,6,7], Marie-Anne Vanderhasselt[5,6], Andreas J. Fallgatter[1,2,3], Ann-Christine Ehlis[1,2,3], David Rosenbaum[1]

[1] Department of Psychiatry and Psychotherapy, Tübingen Center for Mental Health (TüCMH), University of Tübingen, Tübingen, Germany
[2] German Center for Mental Health, Partner site Tübingen, Germany
[3] LEAD Graduate School & Research Network, University of Tübingen, Tübingen, Germany
[4] Department of Psychology, University of Tübingen, Germany
[5] Department of Head and Skin, Psychiatry and Medical Psychology, Ghent University Hospital, Ghent University, Ghent, Belgium
[6] Ghent Experimental Psychiatry (GHEP) lab, Ghent, Belgium
[7] Brain Stimulation and Cognition (BSC) Lab, Department of Cognitive Neuroscience, Faculty of Psychology & Neuroscience, Maastricht University, Maastricht - The Netherlands

Corresponding Author:
Isabell Int-Veen
Calwerstraße 14
72076 Tübingen
Germany
email: isabell.int-veen@med.uni-tuebingen.de

Keywords: Theta Burst Stimulation, stress, Trier Social Stress Test, VLPFC, DLPFC

Supplementary material S1: Inclusion and exclusion criteria of study 1 and study 2

Inclusion criteria:

- age between 18 and 50 years
- normal vision (or appropriate correction)
- right-handedness
- no metal in the skull or brain
- German as native language or very good knowledge of German

Exclusion criteria:

- diabetes mellitus
- renal insufficiency
- untreated hypertension
- history of traumatic brain injury
- cardiac arrhythmia
- acute substance abuse
- adrenal insufficiency
- any acute psychiatric or neurological disorder (including any anomalies in the SCID-Screening (Structured Clinical Interview; First et al., 2015)
- in case of women: pregnancy

Supplementary material S2:

Consort diagram of study 1

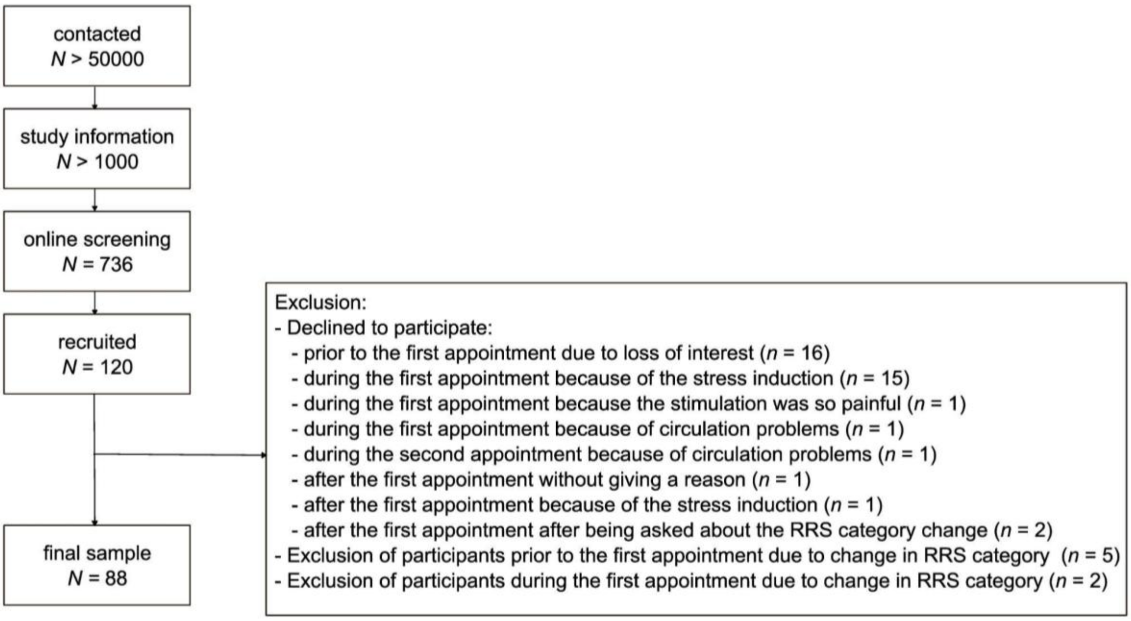

Consort diagram of study 2

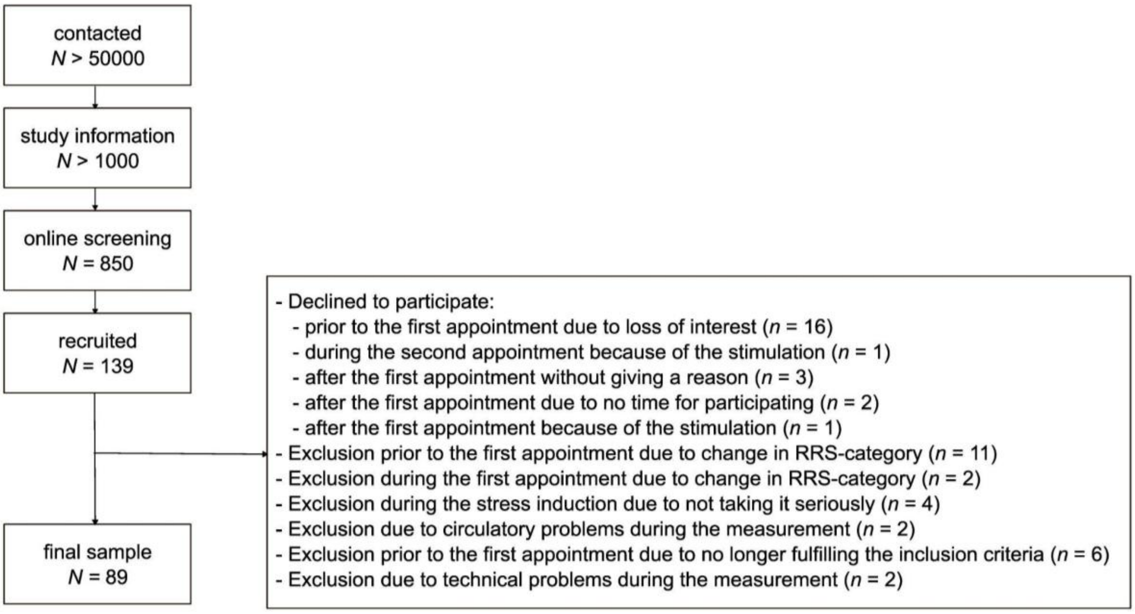

Please note, that we accidentally recruited one additional male high ruminator in the case of study 2. Change in RRS-category: We initially screened low and high ruminators online prior to study inclusion using the RRS (Treynor & Gonzalez, 2003) (low trait ruminators: mean RRS ≤ 1.82 (PR 25); high trait ruminators: mean RRS ≥ 2.36 (PR 64). Please note that these corresponding cut-offs are based on the combined data of 983 participants from prior studies of our group (Rosenbaum et al., 2021; Rosenbaum, Hilsendegen, et al., 2018; Rosenbaum, Thomas, et al., 2018). We noticed substantial changes in RRS scores when participants completed the RRS again at the laboratory which is why we introduced another online assessment of the RRS one week prior to the laboratory appointment. Participants were excluded from the study following the second online questionnaire as well as at the laboratory in case their mean RRS substantially changed either by changing the category or falling between the cutoffs and being closer to the corresponding other category.

Supplementary material S3: Details on the control tasks and Trier Social Stress Test (TSST)

Participants completed two control tasks, each lasting 6 min and consisting of six 40-s trials with 20-s breaks. The first task involved reading number sequences aloud at their own pace (e.g., 1022 minus 17), while the second required mental arithmetic (e.g., repeatedly subtracting 13 from 1007). After each task, stress ratings were assessed. Performance (errors and quantity of items completed) was recorded by the study nurse. In the second task only, participants were asked to restart from the original number upon making an error, mirroring the TSST arithmetic task. Participants then received TBS, followed by another stress rating and a second administration of the SRSRQ. Subsequently, the TSST was conducted. Two experimenters in white lab coats instructed participants to imagine applying for a job and to prepare a 5-min speech about their strengths and qualifications (anticipation phase). After preparation, notes were removed without prior warning, and participants delivered their speech (job interview). If they paused before the 5-min mark, standardized prompts were given to continue. A stress rating followed. Next, participants performed a 6-min arithmetic task consisting of six 40-s trials with 20-s breaks, calculating backward in steps of 13 or 17 from randomized starting points. Errors required participants to restart from the beginning. The experimenters remained neutral and non-responsive throughout the TSST.

Supplementary material S4: Instructions of the TBS

German translation	English translation
Nun folgt die Neurostimulation Ihres Frontalhirns. Dabei laufen je nach Stimulationsart automatisch entsprechende Protokolle ab. Die Stimulation dauert jeweils nur wenige Minuten und die Intensität wird sich langsam steigern, damit Sie sich an das Gefühl gewöhnen können. Die Stimulation wird von den Teilnehmenden als unterschiedlich stark und eventuell auch unangenehm wahrgenommen. Die Stimulation kann sich wie ein Klopfen auf dem Kopf oder wie ein prickeln/ziehen auf der Kopfoberfläche anfühlen. Dabei können Gesichtsmuskeln, wie der Kiefermuskel oder der Augenbrauenheber/Stirnmuskel, direkt oder indirekt mitstimuliert werden und zucken. Bitte schließen Sie während der Stimulation die Augen und versuchen Sie sich möglichst zu entspannen und den Kiefermuskel locker zu lassen, da Verkrampfungen der Muskulatur zu einem unangenehmeren Gefühl beitragen können. Versuchen Sie außerdem, den Kopf ruhig zu halten. Während der Stimulation werden wir die Spule am Stimulationsort auf Ihrem Kopf festhalten.<br><br>Bitte geben Sie Bescheid, wenn während der Stimulation etwas sehr unangenehm oder schmerzhaft sein sollte. Bitte beachten Sie, dass der Abbruch der Neurostimulation jedoch das Ausscheiden aus der Studie zur Folge hat, da die TMS ein wesentlicher Teil der Studie ist.	Now follows the neurostimulation of your frontal brain. Depending on the type of stimulation, corresponding protocols will automatically run. The stimulation lasts only a few minutes each time, and the intensity will gradually increase so that you can get used to the feeling. The stimulation may be perceived by participants as varying in strength and possibly uncomfortable. The stimulation may feel like tapping on the head or like a tingling/pulling sensation on the surface of the head. Facial muscles, such as the jaw muscle or the eyebrow-raiser/forehead muscle, may be stimulated directly or indirectly and may twitch. Please close your eyes during the stimulation and try to relax as much as possible, keeping your jaw muscles loose, as muscle tension can contribute to a more uncomfortable sensation. Also, try to keep your head still. During the stimulation, we will hold the coil in place at the stimulation site on your head.<br>Please let us know if something feels very uncomfortable or painful during the stimulation. Please note that stopping the neurostimulation will result in your exclusion from the study, as TMS is a key part of the study.

Supplementary material S5: Additional figures separately for low and high ruminators

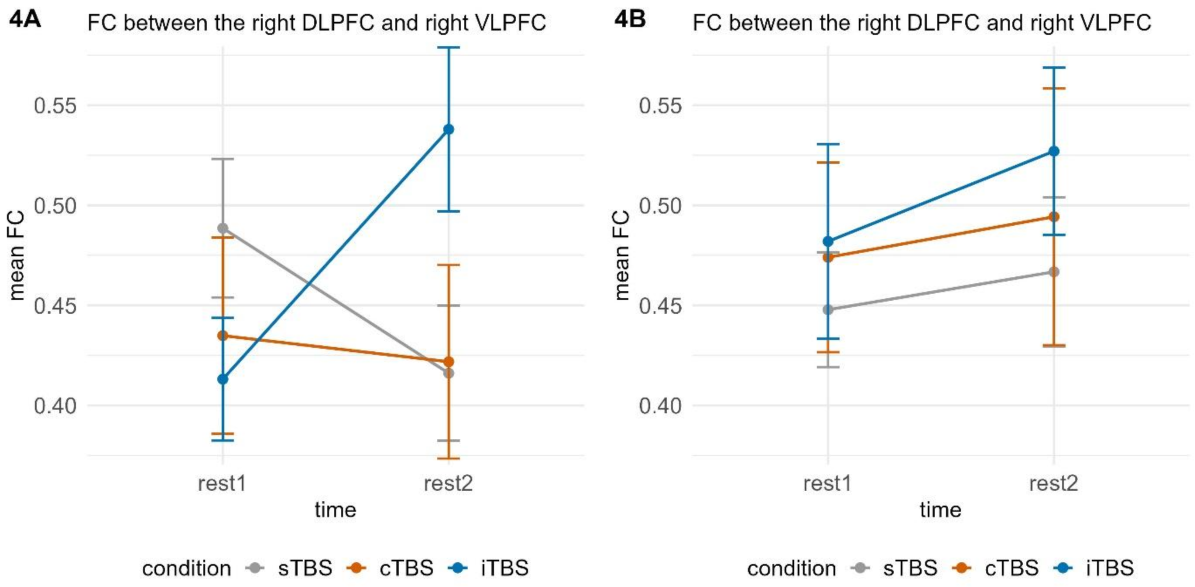

Figure 4. Functional connectivity between the right DLPFC and right VLPFC dependent on time (rest1 vs. rest2) and condition (sTBS vs. cTBS vs. iTBS) for low ruminators (A) and high ruminators (B). Error bars represent ±1 standard error of the mean. sTBS = sham Theta Burst Stimulation, cTBS = continuous Theta Burst Stimulation, iTBS = intermittent Theta Burst Stimulation.

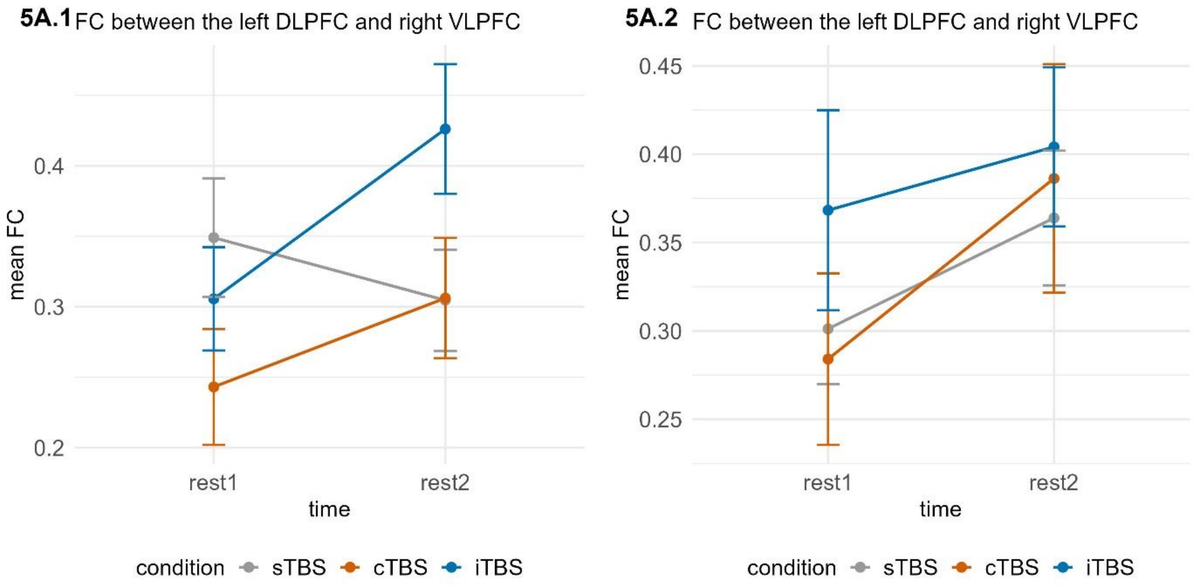

Figure 5A. Functional connectivity between the left DLPFC and right VLPFC for low ruminators (5A.1) and high ruminators (5A.2) dependent on time (rest1 vs. rest2) and condition (sTBS vs. cTBS vs. iTBS). Error bars represent ±1 standard error of the mean. sTBS = sham Theta Burst Stimulation, cTBS = continuous Theta Burst Stimulation, iTBS = intermittent Theta Burst Stimulation.

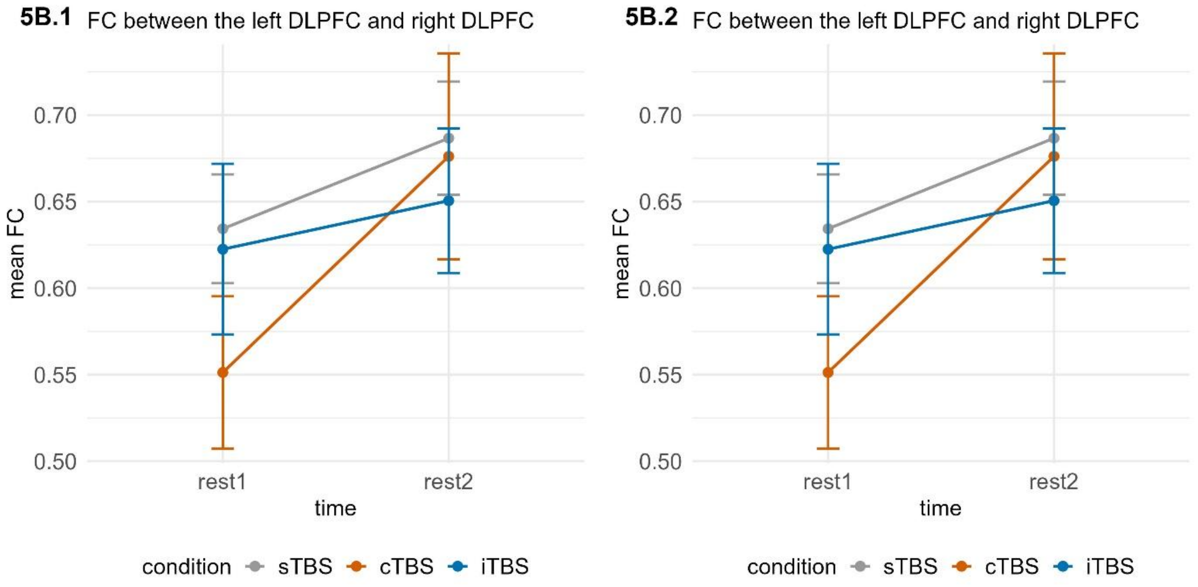

Figure 5B. Functional connectivity between the left DLPFC and right DLPFC for low ruminators (5B.1) and high ruminators (5B.2) dependent on time (rest1 vs. rest2) and condition (sTBS vs. cTBS vs. iTBS). Error bars represent ±1 standard error of the mean. sTBS = sham Theta Burst Stimulation, cTBS = continuous Theta Burst Stimulation, iTBS = intermittent Theta Burst Stimulation.

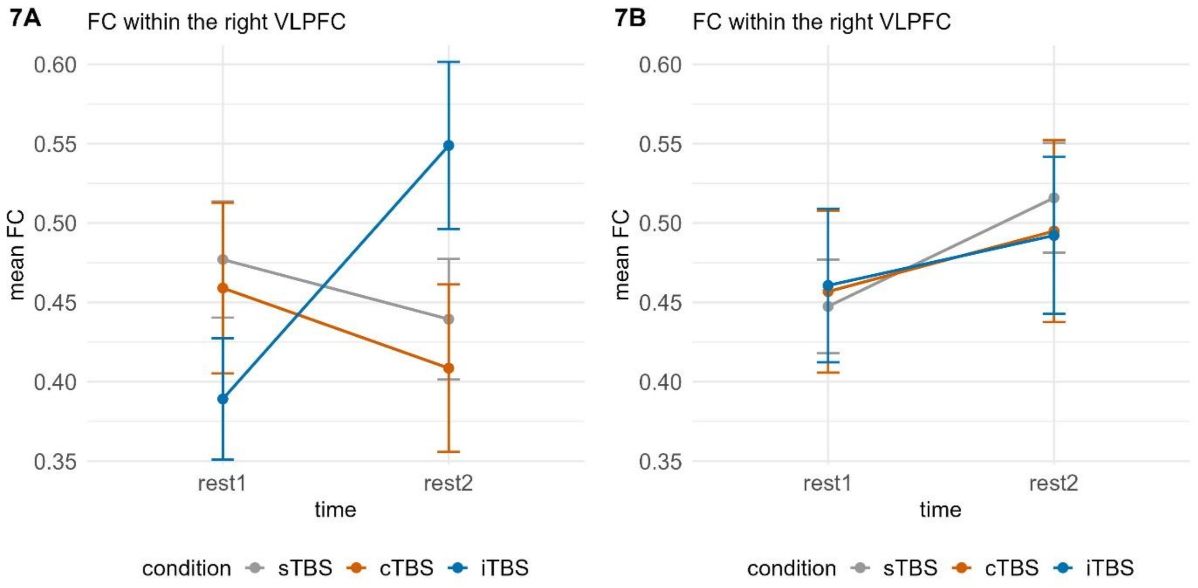

Figure 7. Functional connectivity within the right VLPFC dependent on time (rest1 vs. rest2) and condition (sTBS vs. cTBS vs. iTBS) for low ruminators (A) and high ruminators (B). Error bars represent ±1 standard error of the mean. sTBS = sham Theta Burst Stimulation, cTBS = continuous Theta Burst Stimulation, iTBS = intermittent Theta Burst Stimulation.

References

First, M., Williams, J., Karg, R., & Spitzer, R. (2015). *Structured Clinical Interview for DSM-5 Disorders, Clinical Trials Version (SCID-5-CT)*. American Psychiatric Association.

Rosenbaum, D., Hilsendegen, P., Thomas, M., Haeussinger, F. B., Nuerk, H.-C., Fallgatter, A. J., Nieratschker, V., Ehlis, A.-C., & Metzger, F. G. (2018). Disrupted prefrontal functional connectivity during post-stress adaption in high ruminators. *Scientific Reports*, *8*(1), 15588. https://doi.org/10.1038/s41598-018-33777-1

Rosenbaum, D., Int-Veen, I., Laicher, H., Torka, F., Kroczek, A., Rubel, J., Lawyer, G., Bürger, Z., Bihlmaier, I., Storchak, H., & others. (2021). Insights from a laboratory and naturalistic investigation on stress, rumination and frontal brain functioning in MDD: An fNIRS study. *Neurobiology of Stress*, 100344.

Rosenbaum, D., Thomas, M., Hilsendegen, P., Metzger, F. G., Haeussinger, F. B., Nuerk, H.-C., Fallgatter, A. J., Nieratschker, V., & Ehlis, A.-C. (2018). Stress-related dysfunction of the right inferior frontal cortex in high ruminators: An fNIRS study. *NeuroImage: Clinical*, *18*, 510–517. https://doi.org/10.1016/j.nicl.2018.02.022

Treynor, W., & Gonzalez, R. (2003). Rumination Reconsidered: A Psychometric Analysis. *Cognitive Therapy and Research*, *27*. https://doi.org/10.1023/A:1023910315561
